# Bidirectional Comparisons Revealed Functional Patterns in Interaction between *Salmonella enterica* and Plants

**DOI:** 10.3390/plants13030414

**Published:** 2024-01-30

**Authors:** Min Han, Azhar A. Zarkani, Yongming Duan, Maja Grimm, Jérôme Trotereau, Isabelle Virlogeux-Payant, Adam Schikora

**Affiliations:** 1 Julius Kühn Institute (JKI)—Federal Research Centre for Cultivated Plants, Institute for Epidemiology and Pathogen Diagnostics, Messeweg 11/12, 38104 Braunschweig, Germany; min.han@julius-kuehn.de (M.H.);; 2 INRAE Val de Loire, Université de Tours, L’Unité Mixte de Recherche Infectiologie et Santé Publique (UMR ISP), 37380 Nouzilly, France

**Keywords:** *Salmonella*, Arabidopsis, lettuce, tomato, transcriptome, Tn-Seq

## Abstract

Plants may harbor the human pathogen *Salmonella enterica*. Interactions between *S*. *enterica* and different plant species have been studied in individual reports. However, disparities arising from the distinct experimental conditions may render a meaningful comparison very difficult. This study explored interaction patterns between different *S*. *enterica* strains including serovars Typhimurium 14028s and LT2 and serovar Senftenberg, and different plants (Arabidopsis, lettuce, and tomato) in one approach. Better persistence of *S*. *enterica* serovar Typhimurium strains was observed in all tested plants, whereas the resulting symptoms varied depending on plant species. Genes encoding pathogenesis-related proteins were upregulated in plants inoculated with *Salmonella*. Furthermore, transcriptome of tomato indicated dynamic responses to *Salmonella*, with strong and specific responses already 24 h after inoculation. By comparing with publicly accessible Arabidopsis and lettuce transcriptome results generated in a similar manner, constants and variables were displayed. Plants responded to *Salmonella* with metabolic and physiological adjustments, albeit with variability in reprogrammed orthologues. At the same time, *Salmonella* adapted to plant leaf-mimicking media with changes in biosynthesis of cellular components and adjusted metabolism. This study provides insights into the *Salmonella*-plant interaction, allowing for a direct comparison of responses and adaptations in both organisms.

## 1. Introduction

*Salmonella enterica* is a pathogen continually threating public health. In addition to the typhoidal serovars adapted to humans, non-typhoidal serovars have recently attracted much attention, because of their transmission between animals and different environments, including plants [1]. Non-typhoidal serovars caused multiple outbreaks linked to food, such as meat, nut, fruit, and vegetables [2]. Vegetables such as parsley (*Petroselinum crispum*) [3,4,5], lettuce (*Lactuca sativa*) [5,6,7], and tomato (*Solanum lycopersicum*) [8,9,10] are potential hosts for *Salmonella*. Since many vegetables are ingredients in minimally processed food, *Salmonella* contamination may pose a risk to human health.

*Salmonella* was able to migrate towards plant root or leaf surfaces via chemotaxis [11] using flagella-based motility systems [12,13], and moreover, the colonization can be aided by biofilm formation [4,14]. Successful internalization into plant tissues can potentially shelter *Salmonella* from drought and UV stress. Such internalization may occur via plants’ natural openings, such as cavities after secondary root emergence, or stomata [6,8,15]. During *Salmonella*’s contact with plants, multiple structures on bacterial cytoplasmic membrane were involved. Those included flagellin, a well-known Microbe-Associated Molecular Pattern (MAMP) [16,17], and Type III Secretion Systems (T3SSs) [18,19]. Moreover, synthesis of cellulose and curli was associated with biofilm development, adhesion, and phyllosphere colonization [4,20,21].

When exposed to *Salmonella*, plants may detect its presence by Pattern Recognition Proteins (PRRs)-mediated recognition of certain MAMPs, resulting in Pattern-Triggered Immunity (PTI). These defense events included accumulation of reactive oxygen species (ROS) [16,22], phosphorylation of mitogen-activated protein kinases (MAPKs) [23], regulation of transcription factors [6,23], and activation of plant hormone signaling [9,24,25].

Lettuce and tomato were frequently associated with outbreaks of salmonellosis and were broadly used to study *Salmonella*-plant interaction. In addition, well-annotated genome and many transgenic lines contributed to our understanding of Arabidopsis (*Arabidopsis thaliana*) response to *Salmonella* [23,26,27]. In terms of *Salmonella*, its transcriptional changes during adaptation to plants have also been investigated [6,8,18,27]. In recent years, transposon sequencing (Tn-Seq), a promising technique combining the advantages of mutation and high-throughput sequencing [28], has been applied to investigate conditionally bacterial adaptation, including *Salmonella*. Although much data on *Salmonella*-plant interactions has been established using multiple techniques, the findings are occasionally not consistent, probably due to the use of different *Salmonella* strains or plant species. Other parameters, such as inoculation methods [8] or inoculated leaf region [29], further increases the difficulty to compare differently-sourced results. 

The aim of this study was to investigate and compare the interactions between *Salmonella* and different plants, including Arabidopsis, lettuce, and tomato in a unified manner. We used three *Salmonella* strains: *S. enterica* subsp. *enterica* serovar Typhimurium 14028s (*S.* Typhimurium 14028s), a model strain isolated from poultry; *S. enterica* subsp. *enterica* serovar Typhimurium LT2 (*S.* Typhimurium LT2), a laboratory strain with attenuated virulence due to mutation in *rpoS* [30,31,32]; and *S. enterica* subsp. *enterica* serovar Senftenberg (*S.* Senftenberg), an isolate from basil [33]. We studied and compared *Salmonella* persistence, resulting symptoms, and regulation of pathogenesis-related (PR) proteins encoding genes in Arabidopsis and lettuce leaves using the setup published for tomato [8]. To fill in the gap of tomato global responses to *S.* Typhimurium 14028s, we conducted tomato transcriptome analysis using the inoculation concentration described for Arabidopsis [26] and lettuce [6]. To gain more knowledge on the constants and variants of plants’ responses, we compared their transcriptomes 24 h after challenge with *S.* Typhimurium 14028s. Regarding *Salmonella*, we applied the *S*. Typhimurium 14028s Tn-Seq library [34] to tomato and lettuce leaf-mimicking media and compared the conditionally required *Salmonella* genes. Comparisons of bidirectional sequencing results revealed that plants responded to *S.* Typhimurium 14028s with metabolic and immunological adjustments, while *S.* Typhimurium 14028s adapted to plant leaf-mimicking media with changes in cellular component biosynthesis and adjusted metabolism. This study provides therefore insights into the interaction between *Salmonella* and plants, allowing for a direct comparison of both organisms’ responses and adaptations.

## 2. Results

### 2.1. S. enterica Serovar Typhimurium Strains Persist Better than S. enterica Serovar Senftenberg Strain in Plant Leaves

**Figure 1:**
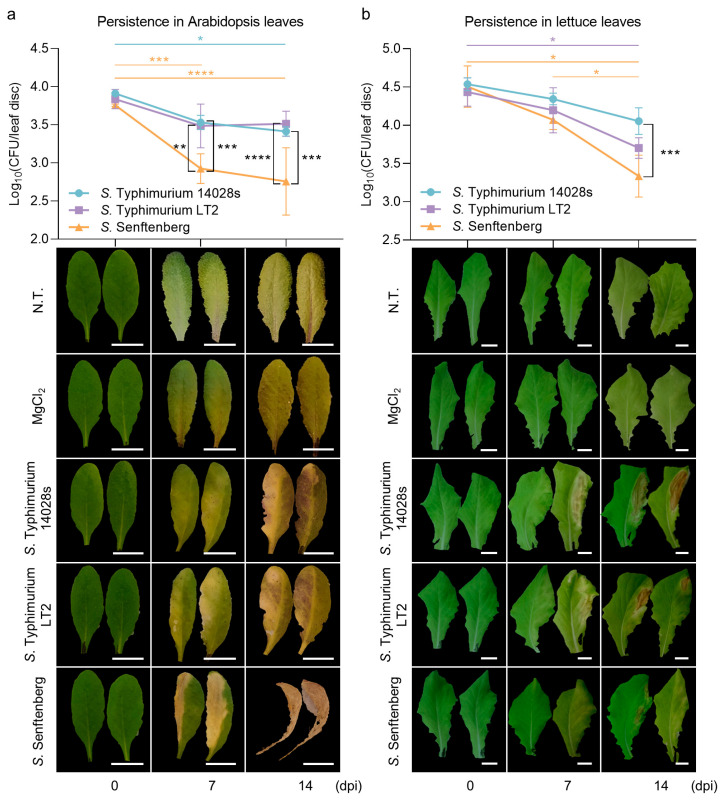
*Salmonella* persistence and resulting symptoms in Arabidopsis (**a**) and lettuce (**b**) leaves. *Salmonella enterica* subsp. *enterica* serovar Typhimurium 14028s (*S.* Typhimurium 14028s), *S. enterica* subsp. *enterica* serovar Typhimurium LT2 (*S.* Typhimurium LT2), and *S. enterica* subsp. *enterica* serovar Senftenberg (*S.* Senftenberg) were used. The error bars represent the standard deviations among three biological replicates. Two-way analysis of variance (ANOVA) with Tukey honestly significant difference (HSD) test was used to determine the differences of one strain at different time points (in corresponding colors) and among three strains at each time point (in black). * *p* < 0.05; ** *p* < 0.01; *** *p* < 0.001; **** *p* < 0.0001. CFU: colony-forming units; dpi: days post inoculation; N.T.: non-treated plants. Bars indicate 1 cm.

Previously published results from tomato leaves revealed steady and comparative persistence of *S.* Typhimurium 14028s and *S.* Typhimurium LT2 over 14 days, but a diminished persistence of *S.* Senftenberg compared to *S*. Typhimurium strains [8]. In order to explore whether similar phenomenon could be observed in Arabidopsis and lettuce, similar experiment was executed. *S.* Typhimurium 14028s, *S.* Typhimurium LT2, and *S.* Senftenberg were individually inoculated, recovered, and enumerated 0 (2 h), 7, and 14 days post inoculation (dpi) (Figure 1). In Arabidopsis leaves, *S.* Typhimurium strains 14028s and LT2 had equivalent persistence capabilities: the colony-forming unit (CFU) numbers dropped slightly 7 dpi but remained steady until 14 dpi, although the changes was not statistically significant at adjacent time points. *S.* Senftenberg, however, displayed significantly inferior persistence as early as 7 dpi (Figure 1a). In lettuce leaves, the numbers of all three strains declined gradually in the course of 14 days (Figure 1b). Slightly different from the situation in Arabidopsis, *S.* Typhimurium LT2 displayed a trend of lower persistence capability than *S.* Typhimurium 14028s, although this difference was not significant. The difference among three strains expanded progressively, and until 14 dpi, *S.* Typhimurium 14028s maintained the biggest population, while *S.* Senftenberg the smallest. These results indicated that *S.* Typhimurium strains have better persistence capabilities than *S.* Senftenberg in tomato, Arabidopsis, and lettuce leaves, although tendencies of each strain throughout the time might fluctuate depending on plant species. However, strain persistence capability was not connected with symptom severity observed for infiltrated leaves. None of those three *Salmonella* strains caused noticeable symptoms on tomato leaves [8]. The Arabidopsis leaves inoculated with *S.* Senftenberg presented the most severe chlorosis and wilt (Figure 1a). However, the lettuce leaves treated with the same strain showed the mildest symptoms (Figure 1b). This phenomenon drew our interest since it indicated the possible involvement of plant defense mechanisms.

### 2.2. S. enterica Strains Regulate the Expression of PR Genes

**Figure 2:**
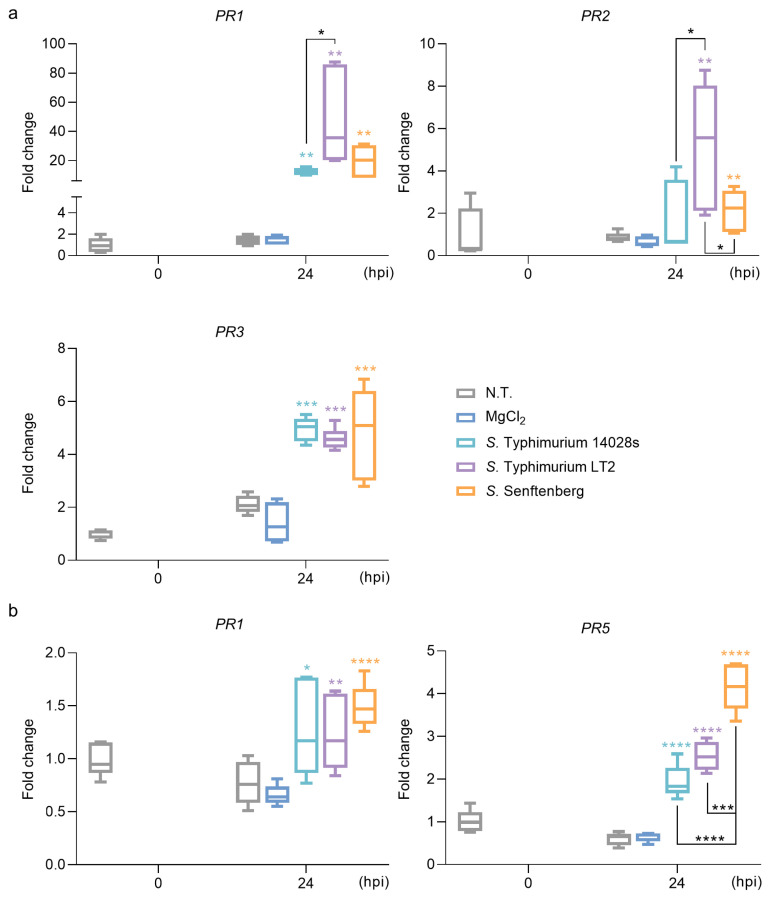
Expression of pathogenesis-related (PR) proteins encoding genes in Arabidopsis (**a**) and lettuce (**b**) challenged with individual *Salmonella* strains (*S*. Typhimurium 14028s, *S*. Typhimurium LT2, or *S*. Senftenberg). Fold changes were calculated by normalizing data to non-treated (N.T.) plants 0 h post inoculation (hpi). Five biological replicates were used. Bar ends represent maximum and minimum values, and the horizontal lines in boxes represent first quartile, median, and third quartile, respectively. Unpaired Student’s *t*-test was used to verify the difference between single *Salmonella* strain and MgCl_2_ treated plants 24 hpi. One-way ANOVA with Tukey HSD test was additionally used to compare the expression in the samples treated with different *Salmonella* strains. * *p* < 0.05; ** *p* < 0.01; *** *p* < 0.001; **** *p* < 0.0001.

Plants activate defense responses when confronted with pathogens, an important example of which is accumulation of PR proteins [35]. *PR* genes in tomato were upregulated in the presence of *S.* Typhimurium LT2 24 h post inoculation (hpi). The response to *S.* Typhimurium 14028s and *S.* Senftenberg were quite gentle and comparable [8]. Genes encoding the apoplast protein PR1 [23,36], plant cell wall β-1,3-glucanases PR2 and chitinases PR3 [23], as well as haumatin-like protein PR5 [37] were chosen in Arabidopsis or lettuce due to previous reports on induction by a *Salmonella* single strain. In order to verify the involvement of *PR* genes in Arabidopsis and lettuce, their expression was determined (Figure 2). We observed upregulation of *PR* genes in Arabidopsis (Figure 2a) and lettuce (Figure 2b) 24 hpi. *S.* Typhimurium LT2 was the strongest inducer in Arabidopsis leaves, similar to tomato, while the strongest lettuce response was induced by *S.* Senftenberg. These findings revealed the upregulation of *PR* genes in plants when they are challenged with *Salmonella*.

### 2.3. Tomato Plants Modulate Their Responses to S. Typhimurium 14028s

In addition to *PRs*, plants may also reprogram other genes while responding to *Salmonella*. To gain a comprehensive understanding on this response, plant transcriptome was evaluated. *S.* Typhimurium 14028s strain was used due to its good persistence and thus potential risk to consumers. The results on Arabidopsis [26] and lettuce [6] were published, while the global response of tomato remained unknown. To close this gap, tomato plants inoculated with *S.* Typhimurium 14028s or 10 mM MgCl_2_ were collected before inoculation, as well as 2, 24, and 48 hpi to track the dynamic of the response.

**Figure 3:**
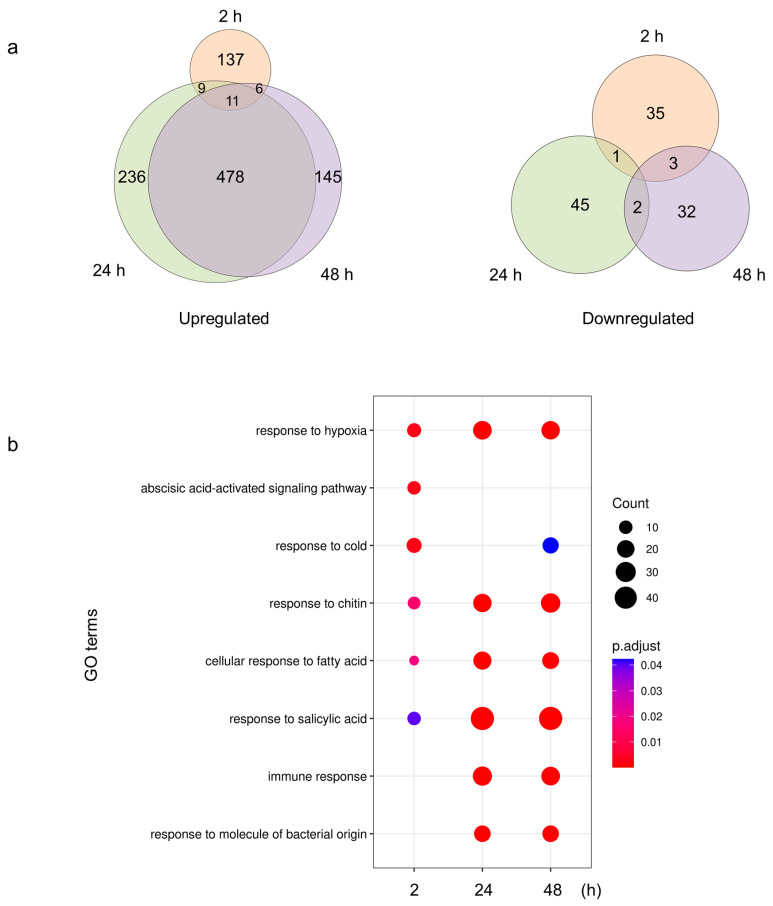
Transcriptional responses of tomato plants to *S*. Typhimurium 14028s 2, 24, and 48 hpi. Four biological replicates were prepared for each treatment. Differentially expressed genes (DEGs) were determined by the threshold of adjusted *p* < 0.05 and |log_2_fold change| > 0.59 using DESeq2. Up and downregulated genes were shown (**a**) and significantly enriched gene ontology (GO) terms were identified (**b**).

Assessment of the differentially expressed genes (DEGs) revealed that tomato plants initiated a transcriptional reprograming already 2 hpi (202 DEGs), followed by a much stronger response 24 hpi (782 DEGs) and 48 hpi (677 DEGs) (Figure 3a, Supplementary Table S1). Notably, 478 upregulated genes were shared between 24 and 48 hpi. Those genes included RECEPTOR-LIKE PROTEIN encoding genes (*RLP6*, *7*, *9*, *12*, *13*, *14*, *27*, *32*, *42*, and *47*), RLP kinase encoding genes (*CRK2*, *3*, *10*, *25*, *26*, and *FLS2*), MITOGEN-ACTIVATED PROTEIN KINASE (MAPK) encoding genes (*MPK4*, *MKP1*, and *MAPKKK5*), and WRKY transcription factors encoding genes (*WRKY6*, *7*, *33*, *40*, *41*, *50*, *51*, *53*, *70*, and *72*). Compared to the number of upregulated genes, only few genes were downregulated (Figure 3a). In order to understand the function of DEGs, enriched gene ontology (GO) terms were assessed (Figure 3b). Responses to multiple abiotic and biotic stimuli were identified 2 hpi, including cold, hypoxia, chitin, fatty acid, abscisic acid (ABA), and salicylic acid (SA). These responses were intensified 24 and 48 hpi, as reflected by increased counts, except for response to cold and ABA signaling. Notably, GO terms related to plant immune response and the response to molecules of bacterial origin were enriched 24 and 48 hpi. These findings suggested that tomato responses to *S.* Typhimurium 14028s are enhanced and specified.

### 2.4. Plants Respond to S. Typhimurium 14028s with Metabolic and Immunological Adjustments

**Figure 4:**
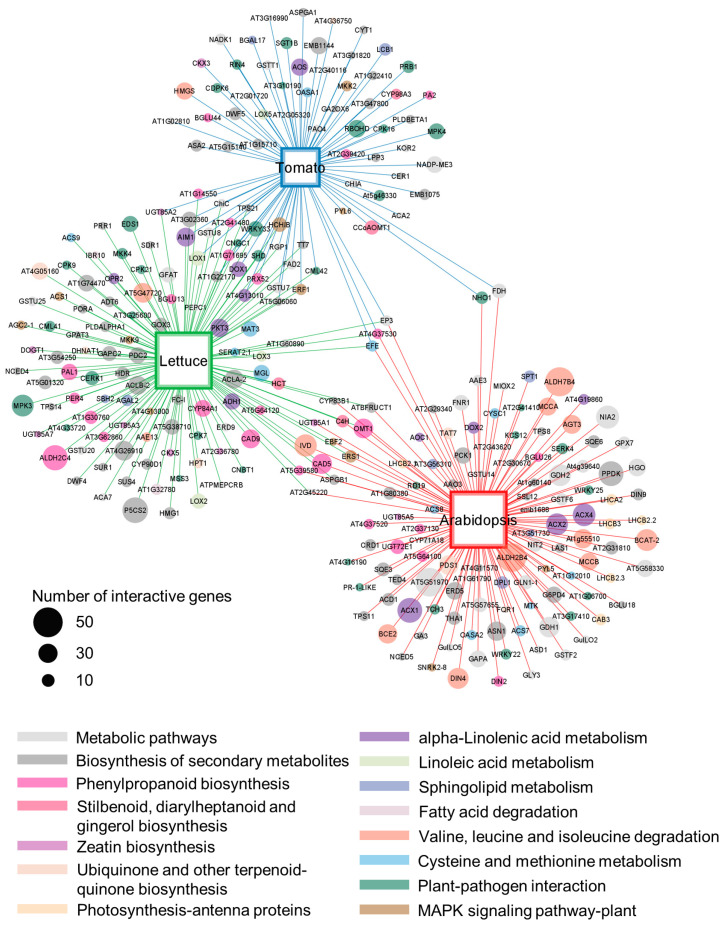
Comparison of transcriptomes in Arabidopsis, lettuce, and tomato inoculated with *S.* Typhimurium 14028s 24 h. Circle color indicates pathway of enriched KEGG as annotated in the legend. Size of the circle represents interactive gene number. Size of the squares indicate number of genes identified in plants. Genes identified in Arabidopsis, lettuce, and tomato are linked in red, green, and blue lines, respectively.

Since *S.* Typhimurium 14028s presence induced strong responses in tomato 24 hpi, we were wondering whether the responses varied depending on plant species at the same time point. For this purpose, the Arabidopsis Information Resource (TAIR) 10 annotated DEGs in Arabidopsis [26], lettuce [6], and tomato (this study) 24 h post inoculation with *S*. Typhimurium 14028s, were extracted and compared (Figure 4 and Supplementary Table S2). Only three orthologues were commonly upregulated in all three plants: *ETHYLENE-FORMING ENZYME* (*EFE*), CHITINASE EP3 encoding gene *EP3*, and a PEROXIDASE encoding gene *AT4G37530*. Many of the upregulated genes were plant species-specific, but pathways that they belong to were similar in all three plants, including metabolic and plant-pathogen interaction processes. One example is calcium signaling in the plant-pathogen interaction. Several calcium signaling genes were regulated in a particular plant: *CPK7*, *9*, *21*, *CML41*, *MSS3*, and *AT3G25600* in lettuce, *CPK16*, *CDPK6*, *RBOHD*, and *AT3G10190* in tomato, as well as *TCH3* and *AT2G41410* in Arabidopsis. Nevertheless, all these genes were included in plant-pathogen interaction group. Those results indicated that plants respond to *S*. Typhimurium 14028s with metabolic and immunological adjustments, albeit with a regulation of different orthologues.

### 2.5. S. Typhimurium 14028s Adapts to Plant Leaf-Mimicking Media with Cellular Component Biosynthesis and Adjusted Metabolism

**Figure 5:**
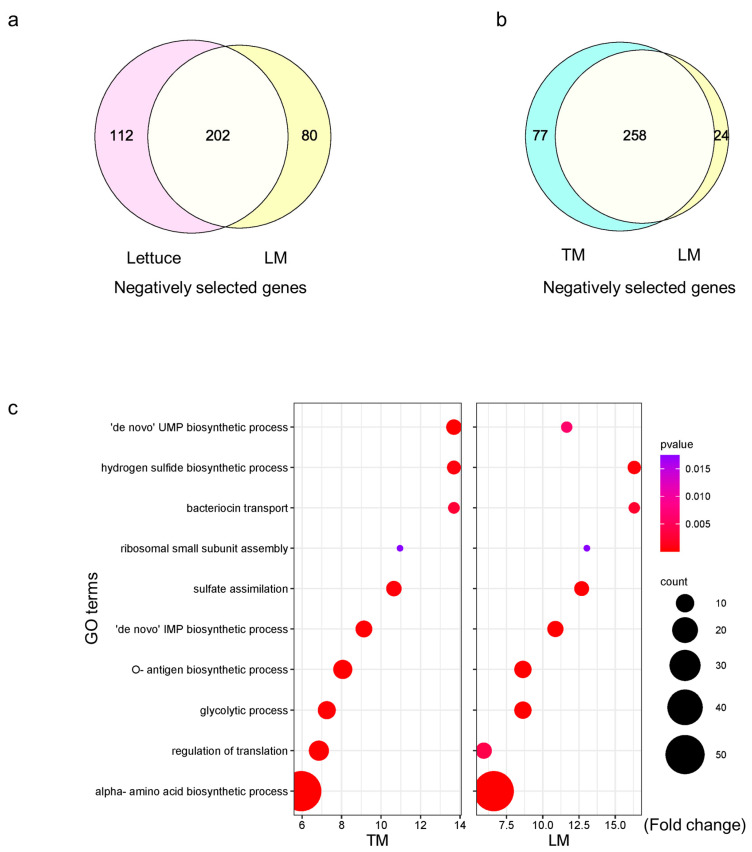
Comparison of genes essential for *S.* Typhimurium 14028s growth in lettuce and tomato-leaf mimicking media (LM and TM, respectively) using *S.* Typhimurium 14028s transposon sequencing (Tn-Seq) library approach. Three biological replicates were prepared for each experimental variant. Both output (24 hpi) and input (control) samples were sequenced. Conditionally essential genes were negatively selected by the threshold of adjusted *p* < 0.05 and log_2_ fold change < −1 using TRANSIT (version 3.2.6) Resampling tool, and were compared (**a**,**b**). Significantly enriched GO terms were determined (**c**). UMP: uridine 5′-monophosphate; IMP: inosine 5′-monophosphate.

To better understand the interaction, we assessed also the bacterial side using *S.* Typhimurium 14028s Tn-Seq library [34]. Given the direct threat to consumers by contaminated vegetables, we focused on *Salmonella* adaptation to tomato and lettuce. Due to the technical difficulty to recover enough *Salmonella* cells from tomato leaves for sequencing, we used tomato leaf-mimicking medium (TM) and lettuce leaf-mimicking medium (LM) as proxies for tomato and lettuce leaves, respectively. Evaluation of the necessity of individual genes was achieved by comparison of the proportion between mutants in input (control) and output samples. The substantial overlap of the negatively selected genes while using inoculated lettuce leaves and LM indicated the suitability of the leaf-mimicking media for a Tn-Seq approach (Figure 5a).

In TM and LM, 335 and 282 genes were negatively selected, respectively, with 258 genes in common (Figure 5b). Those genes were potentially essential for *S.* Typhimurium 14028s adaptation to leaf environment. Only few genes in *S.* Typhimurium 14028s were positively selected (Supplementary Table S3). In the next step, we analyzed GO terms enriched in those gene sets. Results from TM and LM were similar (Figure 5c). Multiple cellular component biosynthesis processes seemed involved, such as ‘de novo’ uridine 5′-monophosphate (UMP) and inosine 5′-monophosphate (IMP) for nucleotides, ribosome small subunit assembly, and O-antigen. In addition, metabolic regulation seemed essential for *Salmonella*’s growth in TM and LM, including sugar utilization and alpha-amino acids biosynthesis. Multiple genes in glycolysis (*pfkA*, *fbaA*, *gapA*, *pgk*, *gpmA*, *eno*, and *pykF*), the first step of glucose catabolism, were identified. In terms of amino acids, biosynthesis of leucine, lysine, proline, threonine, and cysteine was involved in *S*. Typhimurium 14028s growth in both leaf-mimicking media (Supplementary Figure S1). These results indicated that cellular component biosynthesis and metabolic adaptation are required for *S*. Typhimurium 14028s growth in leaf-mimicking media.

## 3. Discussion

According to the European Union One Health 2021 Zoonoses Report, salmonellosis was the second most common zoonosis in 2021 [38]. Moreover, the number of outbreaks associated to vegetables have increased significantly [38]. Many laboratories have reported studies on interactions between *S. enterica* and vegetables. However, due to specific techniques and bacterial/plant species used in individual studies, the results are difficult to compare. In this report, we focused on *S.* Typhimurium 14028s, *S.* Typhimurium LT2, and *S.* Senftenberg, as well as Arabidopsis, lettuce, and tomato. The experiments referred to the setup used in our previous publications [6,8,26], and the results were compared correspondingly.

Serovar was reported as a potential factor leading to *Salmonella* distinct attachment and colonization on plants [11,39]. Better persistence of *S*. Typhimurium strains than *S*. Senftenberg in tomato leaves was reported [8]. This phenomenon was also observed in Arabidopsis and lettuce (Figure 1). Serovar is determined by H-antigen and O-antigen [40]. Arabidopsis leaf wilt was linked to *Salmonella* serovars carrying 1, 3, 19 types of O-antigen, including *S.* Senftenberg [41]. We observed that Arabidopsis leaves inoculated with *S.* Senftenberg exhibited severe wilt (Figure 1a). Nonetheless, plant defenses induced by *Salmonella* may be another factor impacting leaf symptoms. We observed that *S*. Senftenberg induced the strongest *PR* expression in lettuce, followed by *S*. Typhimurium LT2 and 14028s (Figure 2b). Correspondingly, *S*. Senftenberg had lowest persistence and caused the mildest symptoms, whereas *S*. Typhimurium 14028s performed oppositely (Figure 1b). In this process, the virulence of particular strains maybe important, since the virulence plasmid is absent in *S.* Senftenberg [42], and the central regulator encoding gene *rpoS* is mutated S. Typhimurium LT2 [30,31,32].

*S*. Typhimurium 14028s drew our special attention because of its good persistence. Tomato response to *S*. Typhimurium 14028s changed over time. The early response (2 hpi) seemed rather unspecific, including genes responding to multiple stimuli, among others to ABA (Figure 3b). ABA is a general inducer of stomatal closure [43]. Previous reports revealed that *S.* Typhimurium induced transient (2 hpi) stomatal closure in both Arabidopsis and lettuce [44]. Whether those responses are ABA-independent [45] or potentiated by ABA remains unclear. The latter may be true in tomato, as indicated by the ABA-activated signaling pathway induced 2 hpi (Figure 3b). Responses at later time points (24 and 48 hpi) were more specific to bacteria (Figure 3b). Surface recognition is mediated by bacterial MAMPs and plant PRRs. FLAGELLIN SENSING 2 (FLS2) is a typical PRR and was activated in Arabidopsis by *Salmonella*’s flagellin (MAMP), resulting in PTI [16]. In tomato leaves, only some of *S.* Typhimurium 14028s cells expressed flagellin at a detectable level, probably as a recognition avoidance strategy [46]. Despite this, tomato *FLS2* was upregulated 24 and 48 hpi. In addition to PTI, plants can activate immunity triggered by bacterial effectors (ETI) via Nucleotide-binding site Leucine-rich Repeats (NLR) receptors [47]. Several ETI-related genes were upregulated in tomato, including *RPM1 INTERACTING PROTEIN 4* (*RIN4*), encoding a host target of effectors, as well as *NON RACE-SPECIFIC DISEASE RESISTANCE 1* (*NDR1*), encoding a protein anchoring RIN4, required for NLR receptor activation.

Similarly to tomato responses, metabolic and immunological adjustments were observed in Arabidopsis and lettuce (Figure 4). However, different plants may regulate particular genes. Only three orthologue genes *EFE*, *EP3*, and *AT4G37530*, were commonly upregulated in Arabidopsis, lettuce, and tomato. These genes contribute to ethylene biosynthesis, chitin degradation, and peroxide elimination, respectively. Although no independent studies of those genes’ roles in plant response to *Salmonella* have been reported, substantial roles of ethylene [48,49], chitinase [50,51], and peroxidase [52,53] in plant response to biotic and abiotic stressors have been widely explored. Signaling of ethylene interacts with jasmonic acid (JA), SA, and other hormones, regulating plant immunity. Chitinase plays an important role in hydrolysis of fungal cell wall, while is also induced by some bacteria, such as *Pseudomonas syringae* pv. *pisi* [54]. Peroxidase regulates plant ROS production and lignin formation. Notably, those processes are connected with each other. For example, particular groups of chitinase and peroxidase are induced by plant hormone signaling pathways [55,56].

Concerning the adaptation of the other partner in the interaction, we applied *S*. Typhimurium 14028s Tn-Seq library to leaf-mimicking media using the dialysis membrane system. As stated, while describing this method [57], this system may omit genes related to surface contact, as well as T3SS apparatus and effectors. However, the overlap of the genes identified in *Salmonella* recovered from LM (*in vitro*) and lettuce (*in vivo*) indicated that the majority of conditionally essential genes was represented (Figure 5a). We discovered high similarity of essential genes in *Salmonella* grown in TM and LM (Figure 5b,c). Some of those genes contribute to carbon metabolism and biosynthesis of alpha-amino acids. Similar findings were obtained using *Salmonella* RNA-Seq approach in the same system [6,8], and in studies of *Salmonella* colonization of tomato roots [18] and fruits [58], as well as adaptation to different temperatures [59], emphasizing the importance of metabolic adaptation for *Salmonella*. Efficient utilization of carbon sources could benefit in adaptation [60]. Recent studies using transgenic strains underlined the importance of *Salmonella* carbon metabolism in its adaptation to tomato leaves [61]. In terms of amino acids, a notable requirement for cysteine biosynthesis was observed in *Salmonella* adaptation to TM and LM (Supplementary Figure S1), as well as in *Salmonella* colonizing tomato fruits [62] and egg white [63]. Aside from transformation of serine, cysteine can be produced through bacterial sulfate assimilation, which converts inorganic to organic sulfur. *Salmonella* incubated in TM and LM required genes involved in sulfate import and assimilation (Supplementary Figure S1), indicating that both pathways are important for its adaptation to leaf-mimicking media.

Taken together, we compared interactions between different *Salmonella* strains and plants in comparable experimental settings, additionally using comparisons among publicly accessible data generated in a similar manner. Through bidirectional sequencing analysis and comparisons, a basic model of interaction between *S*. Typhimurium 14028s and plants was revealed: plants respond to *Salmonella* via metabolic and immunological adjustments, while *Salmonella* utilizes available nutrients. These findings provide understanding of *Salmonella*-plant interaction and may contribute to the development of particular strategies preventing future salmonellosis outbreaks.

## 4. Materials and Methods

### 4.1. Bacterial Strains, Medium Recipes, and Culture Conditions

*Salmonella enterica* subsp. *enterica* serovar Typhimurium 14028s (*S.* Typhimurium 14028s), *S. enterica* subsp. *enterica* serovar Typhimurium LT2 (*S.* Typhimurium LT2), and *S. enterica* subsp. *enterica* serovar Senftenberg (*S.* Senftenberg), all with spontaneous rifampicin resistance (50 μg/mL) [64], and *S.* Typhimurium 14028s transposon sequencing (Tn-Seq) library with kanamycin resistance (50 μg/mL) [34] were used in this study. Bacteria were cultured in LB medium (Carl Roth, Karlsruhe, Germany) for inoculum preparation. Leaf-mimicking media for lettuce (LM) and tomato (TM) were prepared according to the recipes [25% (*v*/*v*) leaf extract, 1 × M9 salts (5×, Sigma-Aldrich Chemie GmbH, Taufkirchen, Germany), 55% (*v*/*v*) sterile distilled H_2_O] [8,64]. Xylose lysine deoxycholate (XLD) agar (Carl Roth, Germany) was used for *Salmonella* enumeration. *Salmonella* was cultivated at 37 °C in LB and at 28 °C in leaf-mimicking media.

### 4.2. Plant Cultivation

Arabidopsis (*Arabidopsis thaliana* Col-0), lettuce (*Lactuca sativa* L. cultivar Magician), and tomato (*Solanum lycopersicum* cultivar Moneymaker) were used in this study. For persistence assay, seeds were pre-germinated and transplanted to Substrate 1 (Klasmann-Deilmann, Geeste, Germany). Plants were grown for three weeks before inoculation of *Salmonella*. To avoid leaf contamination, plants were irrigated from the bottom of trays as needed. For RT-qPCR and transcriptome assays, seedlings were cultivated in a hydroponic system supported with ¼ MS [8]. Plants were incubated under following conditions: Arabidopsis at 22 °C in short-day conditions (8/16 h, light/dark period), lettuce and tomato at 20 °C with 18 h of daylight.

### 4.3. Salmonella Persistence Assay

Three weeks after transplantation, most recently fully spread three leaves of each plant were chosen. Half side of the leaves were infiltrated either with *Salmonella* at a concentration of 10^7^ colony-forming unit (CFU)/mL, or with 10 mM MgCl_2_. Two hours post inoculation (hpi), four 5 mm-diameter leaf discs were cut from the center of each treated leaf, pooled, and homogenized in 10 mM MgCl_2_. The homogenates were serially diluted and dropped on XLD agar plates with rifampicin (50 μg/mL) for *Salmonella* enumeration. Similarly, samples were collected 7 and 14 days post inoculation (dpi). Separate plants were used at different sampling time points. The persistence ability was evaluated by the assessment of CFU number in leaf discs. At the same time, symptoms on leaves were photographed.

### 4.4. RT-qPCR Assay

Plants were spray-inoculated with *Salmonella* (10^8^ CFU/mL) or with 10 mM MgCl_2_. Non-treated plants were used as references. Plants were sampled before inoculation (0 hpi) and 24 hpi and were homogenized using TissueLyser II (QIAGEN, Hilden, Germany). Immediately afterwards, RNA was extracted using the RNeasy Plant Mini Kit (QIAGEN, Germany), and DNA was eliminated using DNase I (QIAGEN, Germany). Quality and quantity of RNA was verified using the NanoDrop 2000 (ThermoFisher SCIENTIFIC, Waltham, MA, USA). Thereafter, cDNA was synthesized from 1 μg of RNA using qScript cDNA Super Mix Kit (Quantabio, Beverly, MA, USA) and replenished with H_2_O to 100 μL. An RT-qPCR reaction system consisted of 10 μL of LUNA Master Mix (New England Biolabs, Ipswich, MA, USA), 4 μL of H_2_O, 0.5 μL of forward and reverse primers (10 μM), and 5 μL of cDNA. The program in BioRad CFX Connect cycler (BioRad, Feldkirchen, Germany) was set to 95 °C for 5 min, followed by 39 cycles of 95 °C for 15 s and 60 °C for 30 s (+plate read). Fold change of pathogenesis-related (PR) proteins encoding gene expression was calculated by normalization to *actin* gene and non-treated samples (0 hpi) using the 2^−△△Ct^ method [65]. Five biological replicates were used. Each replicate included three plants. The primers are listed in Supplementary Table S4.

### 4.5. Tomato Transcriptome Assay

Tomato plants were spray-inoculated with *S.* Typhimurium 14028s (10^8^ CFU/mL) or with 10 mM MgCl_2_. Four biological replicates were prepared for each treatment. Each replicate consisted of three plants. Leaves were collected before the treatment (0 hpi) as well as 2, 24, and 48 hpi. RNA extraction was performed as described above. DNBSEQ Eukaryotic Strand-specific mRNA library was constructed, and 20M paired end (100 bp length) reads per sample was generated on the DNBSEQ platform in Beijing Genomics Institute (BGI, HongKong, China). The reference genome *S. lycopersicum* cv. Heinz 1706 [66] was used for sequence annotation. Read mapping and feature counting of the data analysis was performed within the R package Rsubread [67]. Untreated samples (0 hpi) were used to normalize gene expression of the samples collected 2, 24, and 48 hpi. Differentially expressed genes (DEGs) were identified by adjusted *p* < 0.05 and |log_2_fold change| > 0.59. For functional analysis and comparisons to the published data, the corresponding Arabidopsis Information Resource (TAIR) IDs of identified DEGs were obtained by aligning tomato protein sequences to Arabidopsis (TAIR10) using Protein BLAST https://blast.ncbi.nlm.nih.gov/Blast.cgi (accessed on 10 February, 2023). The best hit with an e-value less than 10^−10^ was used for the following analysis. Gene ontology (GO) term analysis was performed using clusterProfiler [68].

### 4.6. Salmonella Tn-Seq Library Assay

*S.* Typhimurium 14028s Tn-Seq library consists of 1.34 × 10^6^ independent mutants [34]. Sampling details were previously published [57]. Briefly, the library aliquot was thawed and a part was pelleted as input samples (control). The remaining part was diluted to 10^7^ CFU/mL, inoculated to leaf-mimicking media, and sampled 24 hpi (output samples). In terms of in vivo assay, the inoculum was infiltrated into lettuce leaves and sampled 7 dpi. CFU numbers of *Salmonella* in individual samples adequately covered the library density (Supplementary Figure S2). DNA was extracted, digested, ligated to adapters (Supplementary Table S5), and amplified using Illumina sequencing primers as described [34]. Single-read sequencing (75 bp) with an Illumina NextSeq 550 was executed by I2BC sequencing platform (CNRS, Gif-sur-Yvette, France). Three biological replicates were prepared for each experimental variant. Sequences were aligned to *S.* Typhimurium 14028s genome (NCBI ID: NC_016856.1) and plasmid (NCBI ID: NC_016855.1). Conditionally selected genes were determined by the threshold (|log_2_fold change| > 1, adjusted *p* < 0.05) using TRANSIT (version 3.2.6) Resampling. A minus log_2_ fold change means that the inserted mutant takes up a smaller proportion in the output than input, consequently the gene with transposon insertion is essential for bacterial persistence in the tested condition, while a plus value means the opposite. The enriched GO terms were analyzed using GENEONTOLOGY http://geneontology.org/ (accessed on 7 October, 2022) with ‘*Salmonella* Typhimurium’ as the target organism.

### 4.7. Comparison of Plant Transcriptomes

In published data, Arabidopsis [26] and lettuce [6] were inoculated with *S.* Typhimurium 14028s (10^8^ CFU/mL). Transcriptome was analyzed and DEGs 24 hpi were identified. Moreover, lettuce DEGs were annotated with TAIR10 [6]. For comparison, upregulated genes (fold change > 1.5) in Arabidopsis and lettuce were extracted, and compared with those obtained from tomato 24 hpi in this study. KEGG was used to evaluate the involved pathways.

### 4.8. Statistical Analysis

In *Salmonella* persistence assay, two-way analysis of variance (ANOVA) with Tukey honestly significant difference (HSD) test was used to verify the differences of one strain at different time points and among the strains at each individual time point. In RT-qPCR assay, unpaired Student’s *t*-test was used to verify the difference in gene expression between the plants treated with a single *Salmonella* strain and treated with MgCl_2_ 24 hpi. One-way ANOVA with Tukey HSD test was additionally used to evaluate the difference of the leaves inoculated with three *Salmonella* strains. In tomato transcriptome assay, DEGs were identified using DESeq2 [69] by adjusted *p* < 0.05 and |log_2_fold change| > 0.59. In *Salmonella* Tn-Seq library assay, conditionally selected genes were determined by the threshold (|log_2_fold change| > 1, adjusted *p* < 0.05) using TRANSIT (version 3.2.6) Resampling tool. In all experiments in this study, at least three biological replicates were used, and details are provided in individual assays. 

## 5. Conclusions

In conclusion, this study compared interactions between different *Salmonella* strains (*S*. Typhimurium 14028s, *S*. Typhimurium LT2, and *S*. Senftenberg) and different plants (Arabidopsis, lettuce, and tomato) in similar experimental settings. A good persistence of *S*. Typhimurium 14028s in those three plant leaves was observed. During interaction, plant *PR* genes were differentially upregulated by different *Salmonella* strains. Bidirectional sequencing revealed that plants respond to *Salmonella* via metabolic and immunological adjustments, while *Salmonella* utilizes available nutrients. These findings provide further understanding of *Salmonella*-plant interaction.

## Data Availability

Tomato RNA-Seq and *Salmonella* Tn-Seq datasets in this study can be obtained from NCBI Bioproject by accession numbers PRJNA973841 and PRJNA973854, respectively. Source of the Arabidopsis and lettuce transcriptome data is declared in the references [6,26].

## Supplementary Materials

The following supporting information can be downloaded at: https://www.mdpi.com/article/10.3390/plants13030414/s1, Figure S1: Alpha-amino acid biosynthesis genes required for *S*. Typhimurium 14028s growth in leaf-mimicking media; Figure S2: Numbers of *Salmonella* recovered from lettuce leaves and leaf-mimicking media; Table S1: Tomato RNA-Seq; Table S2: Comparing plant genes; Table S3: *Salmonella* Tn-Seq; Table S4: RT-qPCR primers; Table S5: Oligos (Tn-Seq).
