# Supplementary material for: Bidirectional Comparisons Revealed Functional Patterns in Interaction between Salmonella enterica and Plants
Source: Plants (Basel). 2024 Jan 30;13(3):414. doi: 10.3390/plants13030414 (PMC10857149; doi:10.3390/plants13030414)
Supplement: Supplementary file 1 [file plants-13-00414-s001.zip › Han et al., supplementary files_Plants_R1/Han et al., supplementary figures_R1.pdf]

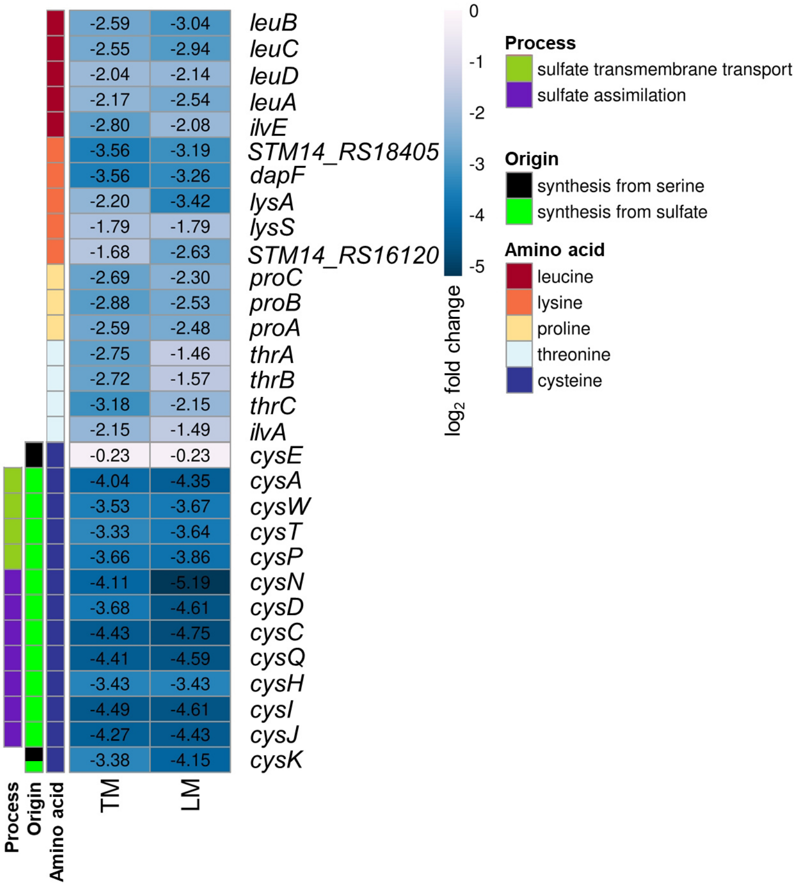

Supplementary Fig. S1 Alpha-amino acid biosynthesis genes required for *S.* Typhimurium 14028s growth in leaf-mimicking media

S. Typhimurium 14028s Tn-Seq library [34] inoculated into TM and LM was sampled 24 hpi. Afterwards, DNA was extracted and sequenced. Values of $\log_2$ fold change of genes were calculated using the TRANSIT Resampling and negatively selected genes (adjusted $p < 0.05$) were determined. A negative value represents essentiality of a gene in the culturing condition. Genes contributing to leucine, lysine, proline, threonine, and cysteine biosynthesis in S. Typhimurium 14028s grown in TM and LM were shown.

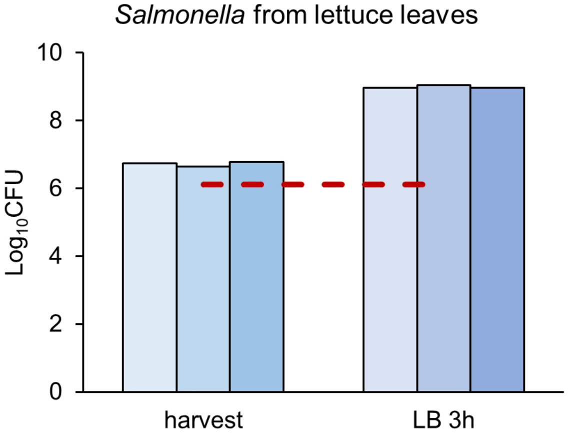

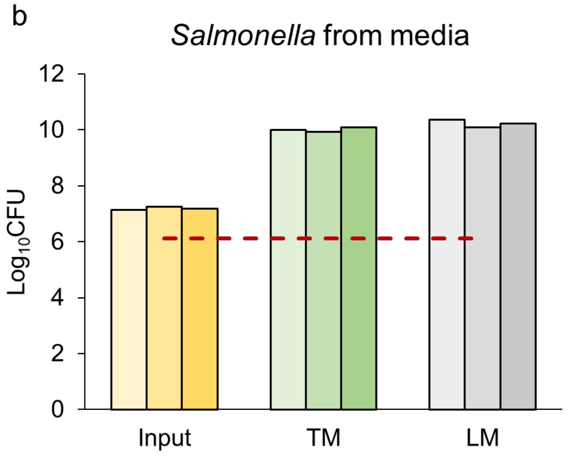

Supplementary Fig. S2 Numbers of *Salmonella* recovered from lettuce leaves and leaf-mimicking media *S.* Typhimurium 14028s Tn-Seq library [34] was inoculated to lettuce leaves and were recovered, enumerated, and enriched in LB medium. The numbers of recovered *Salmonella* cells before and after 3 h population enrichment were determined (a). Inoculum was also incubated in the dialysis membrane bags surrounded by lettuce and tomato leaf-mimicking media (LM and TM, respectively). Afterwards, *Salmonella* sampled from LM and TM were enumerated (b). Three biological replicates under each conditions are indicated in gradient colors. Red dotted lines represent the complexity of *S.* Typhimurium 14028s Tn-Seq library [34].
